# Indirect cholecystoduodenal fistula via hepatoduodenal ligament secondary to gangrenous cholecystitis: a case report

**DOI:** 10.1186/s40792-022-01557-9

**Published:** 2022-10-22

**Authors:** Yasunari Fukuda, Toshiya Michiura, Daisaku Ito, Tomohiro Takahashi, Shinji Tokuyama, Hiromu Morishita, Junya Nuta, Yasuaki Miyazaki, Nobuyasu Hayashi, Kazuo Yamabe

**Affiliations:** 1 Department of Surgery, Kinan Hospital, 46-70 Shinjo, Tanabe, Wakayama, 646-8588 Japan; 2 Department of Gastroenterology, Kinan Hospital, Wakayama, Japan

**Keywords:** Cholecystoduodenal fistula, Hepatoduodenal ligament, Gangrenous cholecystitis, Emphysematous cholecystitis

## Abstract

**Background:**

Cholecystoduodenal fistula is an infrequent complication of gallbladder diseases. In the majority of cases, the fistula is formed by direct communication between the gallbladder and duodenum due to gallstone impaction in the gallbladder neck. We herein report a rare case of indirect cholecystoduodenal fistula via the hepatoduodenal ligament secondary to gangrenous cholecystitis.

**Case presentation:**

An 80-year-old woman suspected of having emphysematous cholecystitis by a previous doctor was referred to our hospital for urgent surgery. The initial diagnosis based on additional examinations was gangrenous cholecystitis penetrating the hepatoduodenal ligament. Since she did not complain of signs of peritonitis and was taking an anticoagulant medicine, we avoided surgery and selected percutaneous gallbladder drainage (PTGBD) instead. Contrast imaging of the PTGBD tube and upper endoscopy identified the indirect cholecystoduodenal fistula via the hepatoduodenal ligament. Despite repeated attempts at endoscopic fistula closure using clips, the fistula did not close successfully. We therefore performed laparoscopic cholecystectomy and fistula closure. The postoperative clinical course was uneventful, and she left the hospital on postoperative day 15. The resected gallbladder contained small black stones, and a histological examination revealed gangrenous cholecystitis with no malignant signatures.

**Conclusion:**

We encountered a rare case of indirect cholecystoduodenal fistula via the hepatoduodenal ligament secondary to gangrenous cholecystitis that was successfully treated by laparoscopic cholecystectomy and fistula closure. It is important to recognize the possible formation of indirect cholecystoduodenal fistula in cases of gangrenous cholecystitis penetrating the hepatoduodenal ligament.

**Supplementary Information:**

The online version contains supplementary material available at 10.1186/s40792-022-01557-9.

## Introduction

Cholecystoenteric fistula is an infrequent condition characterized by spontaneous communication between the gallbladder and adjacent gastrointestinal tract secondary to gallbladder diseases, such as cholelithiasis, cholecystitis, and gallbladder carcinoma. The incidence of cholecystoenteric fistula due to cholelithiasis has been reported to range from 0.2% to 0.5% among all the cases of cholecystectomy, with most cases occurring in the duodenum, accounting for 70–80% of all fistulas, followed by the colon and stomach [1–5]. A cholecystoduodenal fistula is basically formed by direct access between the gallbladder and duodenum due to gallstone impaction in the gallbladder neck. Simultaneous operations of cholecystectomy and fistula closure are the mainstay approach to treating the fistula, but laparoscopic approaches remain challenging [1, 3, 5].

We herein report a rare case of indirect cholecystoduodenal fistula via the hepatoduodenal ligament secondary to gangrenous cholecystitis that was successfully treated with laparoscopic cholecystectomy and fistula closure.

## Case presentation

An 80-year-old woman with persistent abdominal pain for 2 weeks visited a previous hospital and was suspected of having emphysematous cholecystitis. She was referred to our hospital for urgent surgery. Although she reported a complaint of right upper quadrant abdominal tenderness and positive Murphy’s sign, she did not show muscle guarding or rebound tenderness.

Her medical history included chronic hepatitis C after achieving a sustained virological response, myocardial infarction after coronary artery bypass graft (CABG) surgery, hypertension, and hyperlipidemia.

**Fig. 1:**
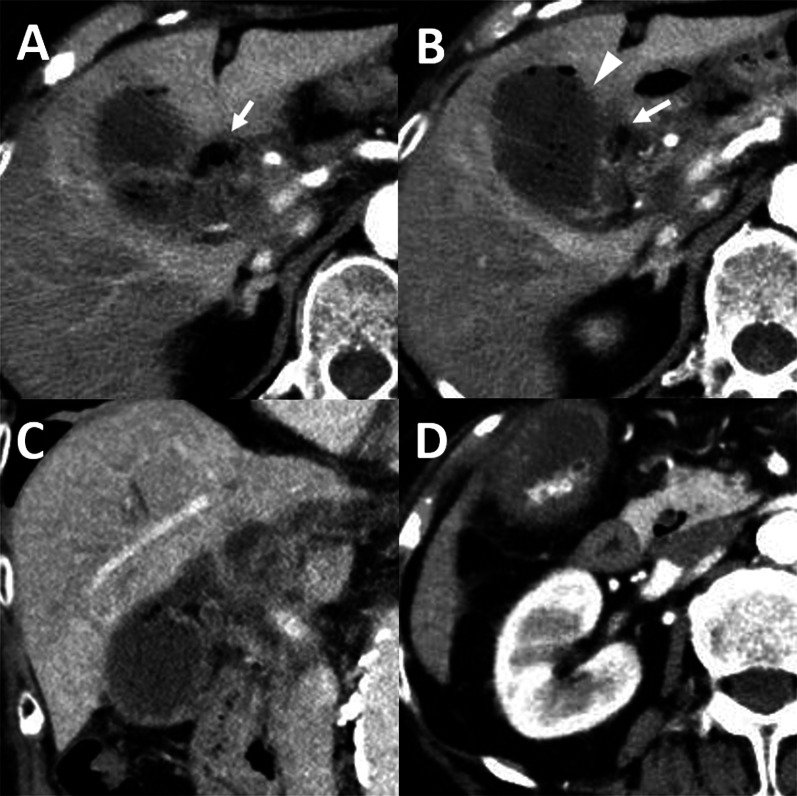
Enhanced CT images of the gallbladder and peri-gallbladder tissues. **A**, **B** CT showed gas formation in the gallbladder lumen and wall that spread to the hepatoduodenal ligament (arrows) and the decreased enhancement of the gallbladder wall (**B** arrowhead). **C** Coronal-view CT showed fluid collection within the hepatoduodenal ligament. **D** CT showed accumulated small gallstones in the fundus of the gallbladder

Enhanced computed tomography (CT) demonstrated gallbladder distention and gas accumulation within the gallbladder lumen and wall that had spread to the hepatoduodenal ligament (Fig. 1A, B and Additional file 1: Video 1, Additional file 2: Video 2). The focally decreased enhancement of the gallbladder wall was also found, suggestive of gangrenous cholecystitis (Fig. 1B). Additionally, fluid collection was observed within the hepatoduodenal ligament (Fig. 1C). Small gallstones were found in the fundus of the gallbladder (Fig. 1D). Abdominal ultrasound findings supported the diagnosis of gangrenous cholecystitis, showing focal perfusion defects on Doppler imaging.

Laboratory test results on admission were as follows: white blood cell count 9300/μL (range: 4000–9500; neutrophils 7140/μL), hemoglobin 10.5 g/dL (range: 11.5–15.3), platelet count 33.6 × 10^3^/μL (range: 150–400 × 10^3^), creatine 0.77 mg/μL (range: 0.5–1.0), amylase 66 IU/L (range: 65–160), aspartate aminotransferase 20 IU/L (range: 11–35), alanine aminotransferase 25 IU/L (range: 5–35), γ-glutamyl transpeptidase 109 IU/L (range: 7–46), alkaline phosphatase 143 IU/L (range: 38–113), total bilirubin 0.8 mg/dL (range: 0.2–1.0), C-reactive protein 5.85 mg/dL (range: 0–0.5), lactate 7.0 mg/dL (range: 4.5–18.0), procalcitonin 0.97 ng/mL (range: < 0.05), carcinoembryonic antigen 2.1 ng/mL (range: < 5.0), carbohydrate antigen 19-9 23.3 U/mL (range: < 37).

Given these findings, the initial diagnosis on admission was gangrenous cholecystitis penetrating the hepatoduodenal ligament. Because two weeks had passed since the symptom onset, she showed no signs of peritonitis, she had controllable inflammation according to blood tests, and she was taking aspirin following CABG surgery, we avoided surgery and selected conservative treatment instead.

**Fig. 2:**
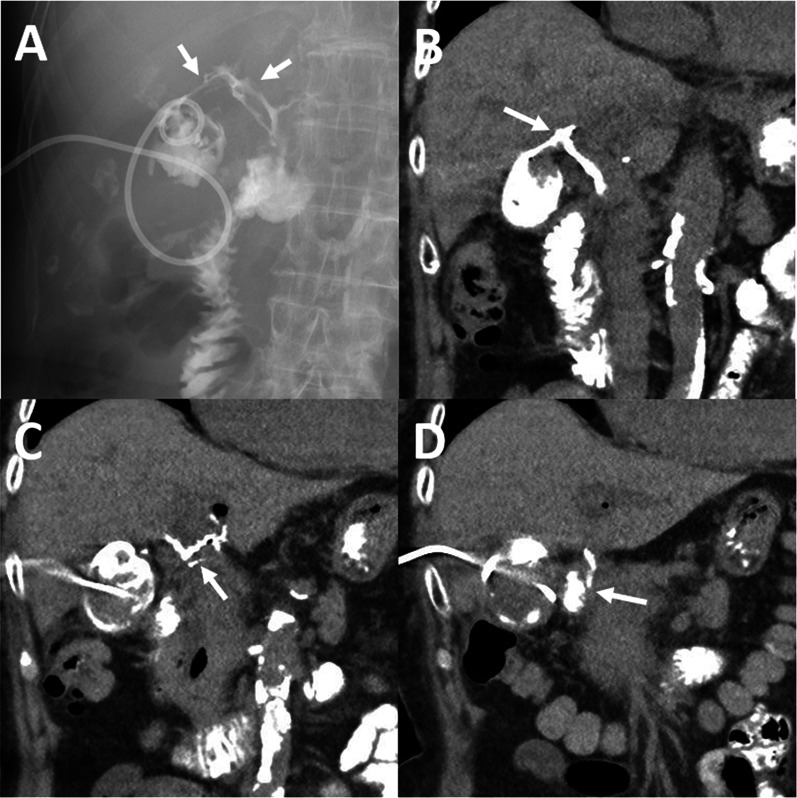
Radiographic contrast examinations. **A** The contrast medium from the PTGBD tube flowed out to the duodenum through the hepatoduodenal ligament (arrows). The cystic duct and extrahepatic bile duct were not contrasted. **B**, **C** CT showed that the contrast medium spread into the hepatoduodenal ligament (arrows). **D** CT showed that the contrast medium in the hepatoduodenal ligament connected to the duodenum (arrow)

**Fig. 3:**
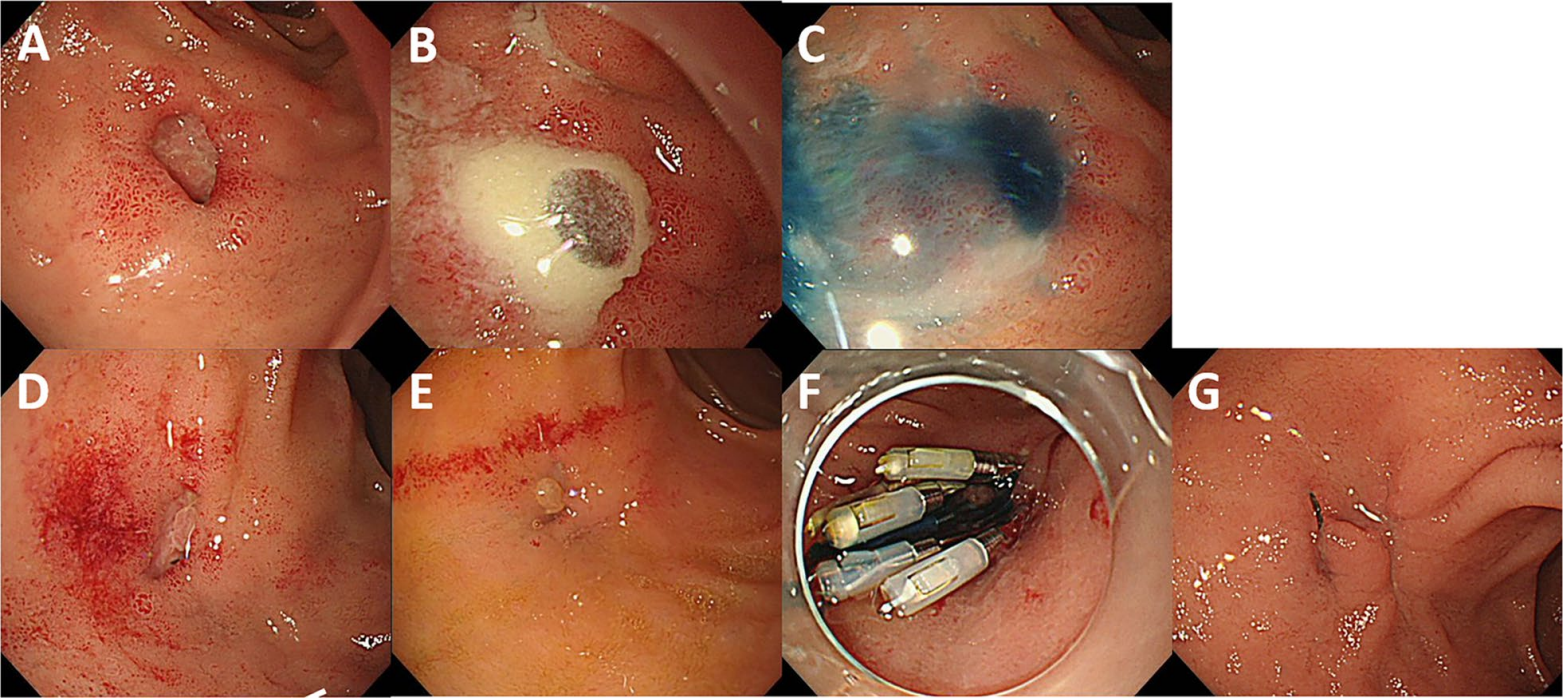
Endoscopic findings. **A** EGD showed a small fistula orifice at the lesser curvature and anterior wall of the duodenal bulb. **B** Pus discharge was found from the orifice. **C** Indigo carmine from the PTGBD tube flowed out to the duodenum. **D**, **E** EGD showed that the fistula had gradually shrunk over time. **E** A granulation polyp was found at the orifice. **F** Although clips were applied endoscopically, the fistula was not successfully closed. Indigo carmine from the PTGBD tube still flowed out to the duodenum. **G** EGD revealed the fistula closure 2 months after surgery

We immediately initiated antibiotic therapy and performed percutaneous gallbladder drainage (PTGBD). Biliary cytology was negative (class II). A radiographic contrast study from the PTGBD tube (Fig. 2A) followed by CT revealed an indirect complicated cholecystoduodenal fistula via the hepatoduodenal ligament (Fig. 2B–D). The cystic duct and extrahepatic bile duct were not contrasted (Fig. 2A). Esophagogastroduodenoscopy (EGD) identified the orifice of the cholecystoduodenal fistula at the lesser curvature and anterior wall of the duodenal bulb (Fig. 3A). Thick pus was discharged from the orifice (Fig. 3B). The instillation of indigo carmine from the PTGBD tube confirmed indirect fistula formation between the gallbladder and duodenum (Fig. 3C).

We next carried out continuous gallbladder irrigation with saline solution to drain the abscess within the fistula. When the local inflammation improved and inflammatory markers were normalized, we ended the irrigation and observed the duodenal fistula using EGD every 7–10 days. Although the size of the fistula hole decreased (Fig. 3D, E), it did not close spontaneously. We therefore attempted to close the fistula endoscopically using clips two times; however, the fistula closure was not successful (Fig. 3F). Therefore, we decided on surgical intervention.

**Fig. 4:**
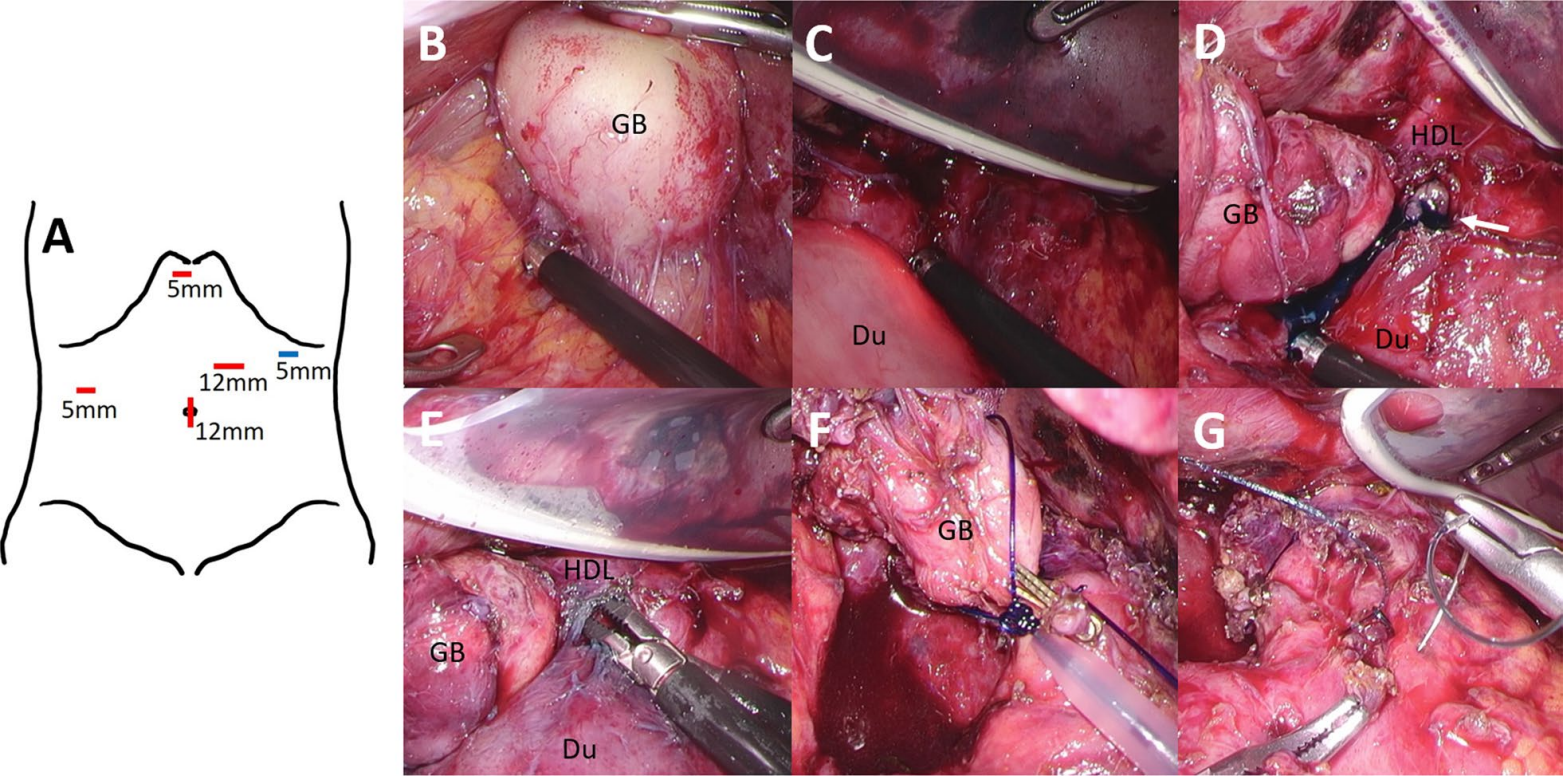
Surgical procedures. **A** Trocar positioning for laparoscopic surgery. Three ports with a camera port were initiated (red lines) and a subcostal port was added intraoperatively (blue line). **B** Inflammation persisted around the gallbladder. **C** We explored the location of the duodenal fistula under endoscopic guidance. **D** Indigo carmine from the PTGBD tube flowed out to the site of the fistula opening (arrow). **E** The fistula led to the hepatoduodenal ligament. **F** Cholecystectomy was performed using the dome-down approach, and the cystic duct was tightened with an Endoloop^®^. **G** Fistula closure at the duodenal side was performed with the running suture technique using V-Loc™. GB: gallbladder, Du: duodenum, HDL: hepatoduodenal ligament

We performed laparoscopic cholecystectomy and fistula closure simultaneously on admission day 57. Under general anesthesia, a laparoscopic procedure was initiated via pneumoperitoneum with an additional three ports based on the French approach of cholecystectomy (Fig. 4A: red lines). Inflammation persisted around the gallbladder (Fig. 4B). First, adhesiotomy around the gallbladder and between the liver and duodenal bulb was performed. A silicone disc was placed from the subxiphoid port to retract the liver while operating between the liver and duodenal bulb, and a left subcostal port was added to assist the laparoscopic procedures (Fig. 4A: blue line). Next, under endoscopic guidance (Fig. 4C), we carefully dissected the tissues around the duodenal bulb followed by transecting the fistula. The fistula opening was corroborated by the instillation of indigo carmine from the PTGBD tube (Fig. 4D). The fistula lad toward the hepatoduodenal ligament (Fig. 4E). We then performed cholecystectomy using the gallbladder bed-first technique (dome-down approach) (Fig. 4F). The cystic duct was tightened with Endoloop^®^ (Ethicon Inc, Cincinnati, OH, USA). We were unable to clearly detect the fistula on the gallbladder side during cholecystectomy. Subsequently, fistula closure on the duodenal side was performed with the running suture technique using V-Loc™ (Medtronic, Minneapolis, MN, USA) (Fig. 4G). No leakage was observed by air leak testing using endoscopy. Overall, no intraoperative complications were observed. The operative time was 4 h and 15 min, and operative blood loss was 22 ml. She had no postoperative complications and was discharged on postoperative day 15. EGD showed the fistula closure 2 months after surgery (Fig. 3G).

**Fig. 5:**
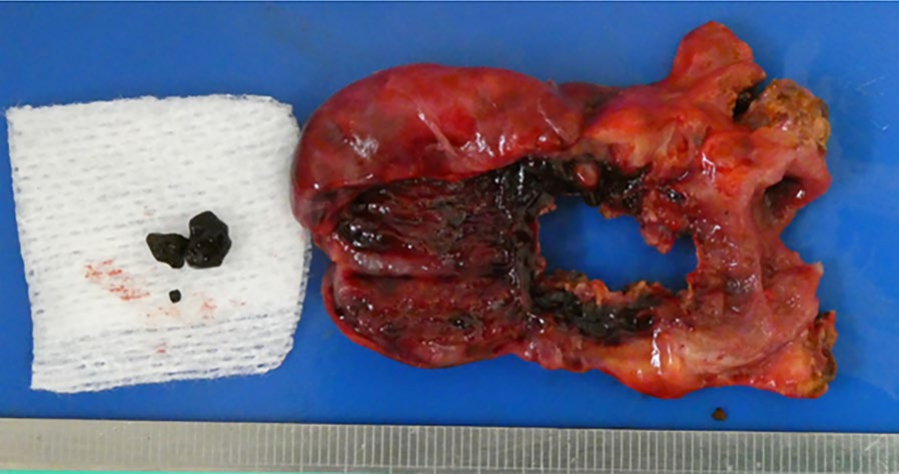
Macroscopic finding of the resected gallbladder. The gallbladder contained several small, black stones. Necrotic changes were found at the gallbladder wall

Macroscopically, the gallbladder contained a couple of small black stones, and its wall was necrotic (Fig. 5). The histological diagnosis was the gangrenous cholecystitis with no malignant findings.

## Discussion

We encountered a case of successful laparoscopic management for indirect cholecystoduodenal fistula via the hepatoduodenal ligament secondary to gangrenous cholecystitis. To the best of our knowledge, this is a first report demonstrating the indirect communication-type cholecystoduodenal fistula.

Cholecystoduodenal fistula typically develops due to the impact of gallstones in the Hartmann’s pouch followed by the subsequent increased pressure inside the gallbladder and inflammatory process that leads to adhesion and fistulous formation to the duodenum. Therefore, the fistula is fundamentally a direct communication between the gallbladder and duodenum. In our series, as a consequence of gangrenous cholecystitis that penetrated the hepatoduodenal ligament and induced stricture of the cystic duct, pressure inside the gallbladder was predominantly relieved into the ligament side, which may be involved in the development of indirect access to the duodenum.

Symptoms associated with cholecystoduodenal fistula are non-specific and variable dependent of the extent of inflammation. In some cases where the migration and impaction of large stones within the gastrointestinal tract causes gastric outlet obstruction (known as Bouveret syndrome [6]) or small bowel obstruction, symptoms related to ileus may occur, including abdominal distention, nausea, and vomiting [7]. In addition, symptoms of hematemesis or melena as a consequence of stone erosion of the gastrointestinal wall can prompt the performance of endoscopy, which makes it possible to detect the fistula [1, 8].

However, a preoperative diagnosis with non-specific symptomatic cholecystoduodenal fistula is challenging, and most reported cases are diagnosed intraoperatively. Based on a large cholecystectomy cohort study, only 26.1% of patients were found to have cholecystoduodenal fistula preoperatively [5]. That study also denoted that, in addition to a history of repeated episodes of cholecystitis, air in the gallbladder and pneumobilia as well as an ill-defined border between the gallbladder and duodenum on ultrasound and CT are important clues for the detection of cholecystoduodenal fistula preoperatively [5]. Pneumobilia was not relevant in the present case, probably because of the stricture or obliteration of the cystic duct. However, gas seen in the gallbladder and the hepatoduodenal ligament were crucial findings prompting additional examinations, such as a contrast study and endoscopy, which are capable of distinguishing complicated gangrenous cholecystitis with an indirect cholecystoduodenal fistula. When encountering emphysematous cholecystitis, we should always keep in mind that air is made not only by gas-producing bacteria but also by fistula formation between the biliary system and gastrointestinal tract.

The principle strategy for the treatment of cholecystoenteric fistula, including cholecystoduodenal fistula, is cholecystectomy and fistula closure. With regard to fistula closure, there are two approaches: surgical and endoscopic approaches. Surgical stapling or hand sewing is used to close fistula surgically. However, a laparoscopic approach has already achieved wide acceptance in the surgical treatment of cholecystoenteric fistula, with increased rates of successful completion over time, and a more than 80% success rate has been reported according to the recent large-scale cohort studies, although it still requires considerable expertise to perform [1, 3, 5]. In the current case, relatively easy access to the cholecystoduodenal fistula that led to the lesser curvature and anterior wall of the duodenal bulb allowed us to complete the laparoscopic surgery. Intra-operative endoscopy was also an important adjunct to this surgery. In contrast, endoscopic application of clips (through-the-scope clips or over-the-scope clips), a stent, and fibrin glue would be viable alternatives to surgery for fistula closure [9–11]. In the present case, as we were considered likely to have difficulty detecting and opening the fistula during surgery, we tried to close the fistula with non-surgical management using endoscopic clipping. However, endoscopic management failed, likely because the tissue surrounding the fistula hole had already hardened due to long-term inflammation.

## Conclusion

In conclusion, we report a rare case of indirect cholecystoduodenal fistula via the hepatoduodenal ligament secondary to gangrenous cholecystitis successfully treated with laparoscopic cholecystectomy and fistula closure. In contrast to direct communication-type fistula, it deems extremely difficult to recognize the presence of indirect fistula intraoperatively. In this regard, a preoperative diagnostic awareness of the indirect cholecystoduodenal fistula is important in cases of gas accumulation within the hepatoduodenal ligament, as this type of fistula demands particularly advanced therapeutic strategies.

## Supplementary Information

**Additional file 1: Video 1.** Axial-view CT images showing the gallbladder and peri-gallbladder tissues.

**Additional file 2: Video 2.** Coronal-view CT images showing the gallbladder and peri-gallbladder tissues.

## Data Availability

The data that support the findings of this study are available from the corresponding author upon reasonable request.

## Abbreviations

CT: Computed tomography
PTGBD: Percutaneous gallbladder drainage
EGD: Esophagogastroduodenoscopy
